# Simulation Analysis of Impulsive Ankle Push-Off on the Walking Speed of a Planar Biped Robot

**DOI:** 10.3389/fbioe.2020.621560

**Published:** 2021-01-12

**Authors:** Qiaoli Ji, Zhihui Qian, Lei Ren, Luquan Ren

**Affiliations:** ^1^Key Laboratory of Bionic Engineering, Jilin University, Changchun, China; ^2^School of Mechanical, Aerospace and Civil Engineering, University of Manchester, Manchester, United Kingdom

**Keywords:** biped robot, 2D walking, ankle push-off, ankle torque, walking speed

## Abstract

Ankle push-off generates more than 80% positive power at the end of the stance phase during human walking. In this paper, the influence of impulsive ankle push-off on the walking speed of a biped robot is studied by simulation. When the push-off height of the ankle joint is 13 cm based on the ground (the height of the ankle joint of the swing leg) and the ankle push-off torque increases from 17 to 20.8 N·m, the duration of the swinging leg actually decreases from 50 to 30% of the gait cycle, the fluctuation amplitude of the COM (center of mass) instantaneous speed of the robot decreases from 95 to 35% of the maximum speed, and the walking speed increases from 0.51 to 1.14 m/s. The results demonstrate that impulsive ankle push-off can effectively increase the walking speed of the planar biped robot by accelerating the swing leg and reducing the fluctuation of the COM instantaneous speed. Finally, a comparison of the joint kinematics of the simulation robot and the human at a normal walking speed shows similar motion patterns.

## Introduction

Some studies of ankle push-off with respect to stability, energetic efficiency and disturbance rejection have been conducted using simulation models and real biped robots. However, the effect of ankle push-off on the walking speed of bipedal robots has not been studied deeply. In this paper, the influence of impulsive ankle push-off on the walking speed of a biped robot is studied via a simulation method. We first review past work on the role of ankle push-off during human walking and some studies of ankle push-off in simulation models and real biped robots, followed by our simulation model and controller. At last, the results and discussion are presented.

### Ankle Push-Off in Human Walking

Ankle push-off is the positive power or work generated by the plantarflexor muscles and tendons about the ankle joint at the end of the stance phase during the step-to-step transition in human walking (Zelik and Adamczyk, 2016). Ankle push-off occurs at 45–65% of the gait cycle (Zelik and Kuo, 2010). Due to its significant role in human locomotion, ankle push-off has been studied in biped robots in many previous studies. Dean and Kuo (2009) presented that the advantage of the ankle push-off function is to reduce both the velocity of the COM (center of mass) at the heel strike and the energy loss caused by the collision (Dean and Kuo, 2009). These researchers also suggested that the main function of ankle push-off is to redirect the COM velocity during the step-step transition (Kuo and Donelan, 2010). Some studies have shown that ankle push-off primarily contributes to powering leg swing by accelerating the trailing limb (Winter and Robertson, 1978; Meinders et al., 1998). In summary, (Zelik and Adamczyk, 2016) presented a unified perspective of ankle push-off in human walking and suggested that ankle push-off not only accelerates the swing leg but also accelerates the COM (Zelik and Adamczyk, 2016). On one hand, the mechanism of the peak power generated by ankle push-off was revealed and the results show that the impulsive ankle push-off quickly powers leg swing during human walking (Lipfert et al., 2014).

On the other hand, the study shows that the COM work exhibits a similar timing and magnitude with the ankle push-off work of the trailing limb. Furthermore, the ankle push-off work of the trailing limb was estimated to contribute more than 80% of the positive COM push-off work (Zelik and Adamczyk, 2016). Furthermore, previous studies demonstrated that the reduced ankle push-off contributes to increased metabolic energy expenditure accompanying ankle impairments (Collins and Kuo, 2010). Moreover, the transition work increased if one or both legs did not push-off with optimal coordination (Soo and Donelan, 2011). Thus, ankle push-off provides substantial work and contributes to the COM acceleration.

### Related Works of Ankle Push-Off in Biped Robots

Due to the importance of ankle push-off during human walking, researchers have explored the energy efficiency, stability, and disturbance rejection by using ankle push-off in simulation models and real bipedal robots. Simulation studies of a simple walking model show that an idealized impulsive ankle push-off of the trailing limb presents an energy-saving effect before the leading leg heel-strike (Kuo, 2002). The Cornell biped robot has partially realized this concept and has become the most efficient actuated bipedal walker in existence (Collins et al., 2005). In addition to energy efficiency, the effects of ankle push-off on the stability and disturbance rejection have also been studied in biped robots. The simulation model of a compass walker has acquired stable periodic motion based on energy regulation using a combination of ankle push-off control and foot placement control (Bhounsule, 2015). Kim et al. proved that the discrete control of ankle push-off is very effective at recovering from two different disturbances (random changes in the ground height and lateral impulses) at slow and normal speeds in a 3D simulation of bipedal walking (Kim and Collins, 2017). To further explore how ankle actuation influences energy use and disturbance rejection in a real biped robot, Hobbelen et al. designed the planar biped robot “Meta” with two actuated ankle joints. It proved that modulating the ankle push-off torque based on feedback from the leading leg angle with respect to gravity can improve the disturbance rejection of the prototype by at least 60% compared with the situation without ankle push-off feedback. Additionally, ankle push-off feedback increases the energy efficiency of a walker, while this finding has only been obtained in simulation models (Hobbelen and Wisse, 2008a).

### Related Works of Effect of Ankle Push-Off on Walking Speed of Biped Robots

The walking speed is also an important aspect of the versatility of biped robots. As we know humans are able to perform a variety of walking speeds with high efficiency, low energy consumption, and good disturbance rejection. The maximum walking speed of humans is 4.6 m/s, and the dimensionless speed is approximately 1.4–1.5 (Sreenath, 2011). Compared with human walking speed, there is still a certain gap for bipedal robots. To further understand the mechanism of human walking, researchers have tried to improve the performance of autonomous bipedal robots (Hobbelen and Wisse, 2008b) and study the problem that how to increase the walking speed of bipedal robots. Grizzle's team designed a series of planar biped robots, including “Rabbit” and “Mabel.” The former obtains a maximum walking speed of 1.2 m/s, which is driven by a controller that is designed with the virtual constraint and hybrid zero dynamics (HZD) (Chevallereau et al., 2003). The latter implements a biped with mechanically adjustable series compliance (BiMASC) structure to increase the walking speed (Hurst et al., 2007; Hurst and Rizzi, 2008; Grizzle et al., 2009). After that, Hurst et al. designed an underactuated robot “ATRIAS” based on the mass-spring model, and the robot obtained a maximum speed of 2.5 m/s (Ramezani et al., 2014; Hubicki et al., 2018). However, compared with humans, the biped walkers “Rabbit,” “Mabel” and “ATRIAS” have no ankle joints. Therefore, the walking gait of these biped robots may not be effective at providing an understanding of human walking. Then, some researchers have attempted to study human-like biped robots. The multi-contact biped robot “AMBER” was designed and obtained a stable walking gait when using human walking data (Zhao et al., 2017). The biped robot “DURUS” was designed by Hereid et al. and realized 2D dynamic walking by using an HZD gait optimization method. The robot's walking speed was approximately 0.3 m/s, and its energy consumption was approximately 1.33 (Hereid et al., 2018). The biped robots “AMBER” and “DURUS” have actuated ankle joints. However, the walking speeds of “AMBER” and “DURUS” have not significantly increased. Thus, some researchers attempt to use new methods to increase walking speed of biped robot, including actuated ankle push-off and impulsive ankle push-off.

#### Actuated Ankle Push-Off

The bipedal robot “RunBot” using sensing-driven neuron controllers and real-time online learning methods can walk at a speed of 3.5 times the leg length per second (0.8 m/s) after several minutes of learning (Geng et al., 2006; Geng, 2014). Another study regarding increasing walking speed is based on virtual slope walking. Specifically, the planar biped robot “Stepper-2D” was designed by Dong et al. (2011) and can realize fast walking and walking speeds up to 4.48 times the leg length per second (1.65 m/s) (Dong et al., 2011). The walking speeds of “RunBot” and “Stepper-2D” increased using the above methods. However, the biped robots “RunBot” and “Stepper-2D” have passive ankle joints or only point feet. The biped robot without ankle joint or with passive ankle joint is difficult to provide propulsive force by using ankle push-off, thus the biped robot lack ankle joint just as effectively as those that lack ankle push-off. These previous researches all have shown that the lack of actuated ankle push-off leads to a more severe acceleration-and-brake effect for biped robots, which has become a disadvantage of passive ankle walkers during fast walking (Geng, 2014). It suggests that actuated ankle push-off could be a very important method for fast walking for biped robots.

#### Impulsive Ankle Push-Off

Furthermore, Pratt found that the main factors affecting the walking speed of bipedal robots are the stride length and swing time (Pratt, 2000). Moreover, the magnitude of ankle push-off simultaneously affects both the step length and walking speed, and the simulation shows that walking speed roughly increases with the square root of the push-off magnitude (Dean and Kuo, 2009). Hobbelen et al. explored the effect of the amount of ankle push-off, upper body pitch and step length on the walking speed of the Limit Cycle Walker “Meta,” and the results show that adjusting the ankle push-off or upper body pitch can increase the walking speed. This prototype has obtained walking speeds ranging from 0.24 to 0.68 m/s (the dimensionless speed is represented by the normalized Froude number *Fr* = 0.1 to 0.28). However, due to the passive knee joint of “Meta,” a further increase in the walking speed is limited (Hobbelen and Wisse, 2008b). In addition, the ankle joints of “Meta” are actuated by electric DC motors, which cannot achieve impulsive push-off in practice. Thus, the ankle push-off is still different from that of humans, and this may be one of the key reasons limiting the improvement of walking speed. Therefore, obtaining impulsive ankle push-off could be another significant method to increase the walking speed of biped robots.

Due to the factor that passive ankle joints and ankles actuated by motors cannot generate sufficient impulsive ankle push-off torques. The pneumatic cylinder may be the method to generate explosive force. The simulation has been recognized as an important research tool in robotics and can be used for the kinematics and dynamics analysis of robots, for off-line programming, to design different control algorithms, etc. (Žlajpah, 2008). Thus, this paper aims to firstly study the effect of impulsive ankle push-off on the walking speed of planar bipedal robots via a simulation. The simulation may provide a reference and support for the biped robot prototype.

## Methods

### Model

**Figure 1:**
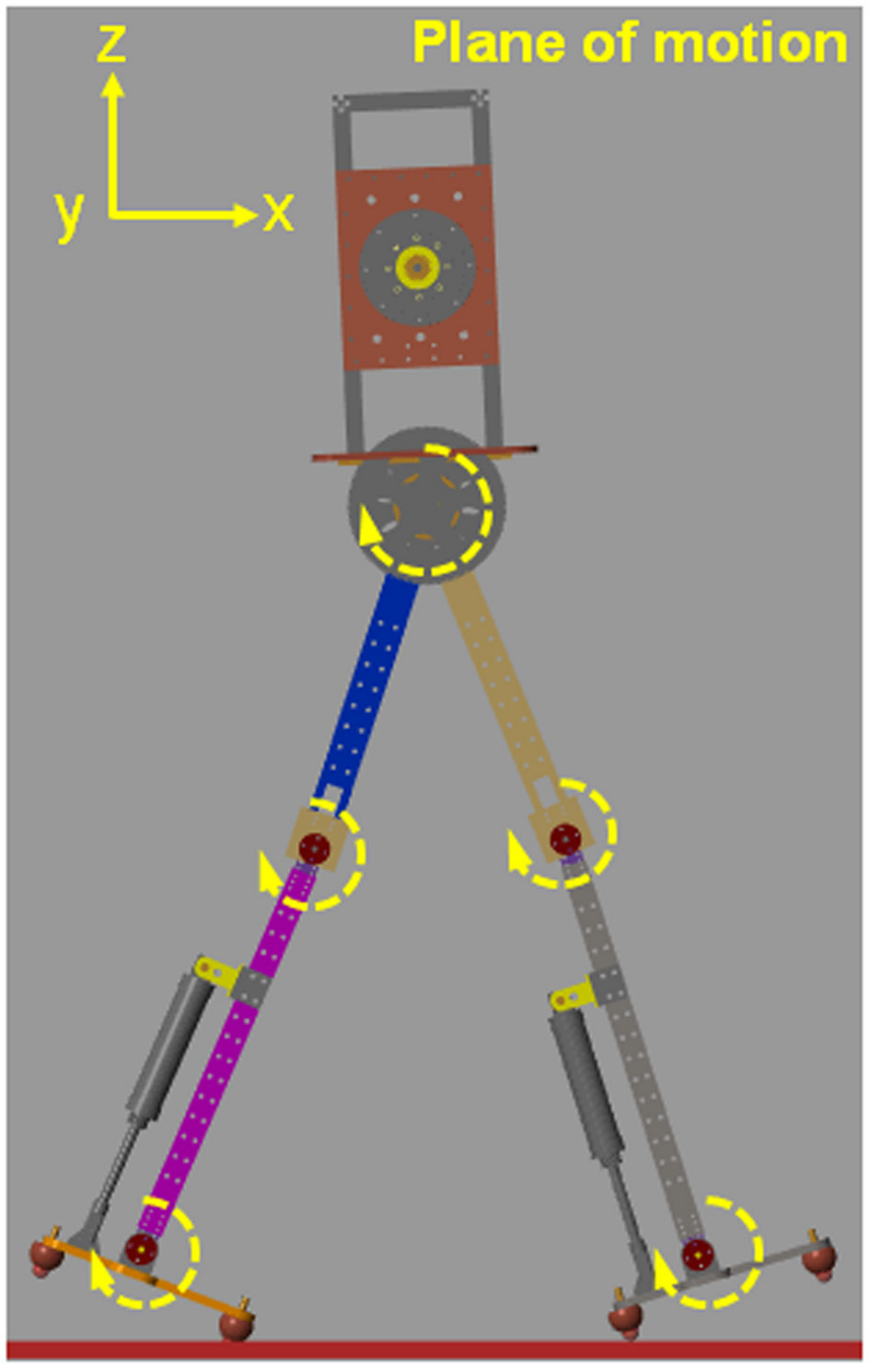
The simulation model of bipedal robot. The simulation model is restricted in the plane of motion.

In this study, we designed and constructed a simulated model of planar biped robot, as shown in Figure 1. The parameters of the mass distribution and mechanical structure of the simulation model are shown in Table 1. The simulation model is used to explore the effect of impulsive ankle push-off on the walking speed by using the MATLAB/Simulink Simscape multibody toolbox. The biped robot has a torso and two legs. Each leg has three degrees of freedom. To constrain the bipedal robot to walk only in the sagittal plane, three degrees of freedom (two prismatic and one revolute, see Figure 1) were added to the robot torso and the earth of the simulation environment. The 3D contact model (a block named Sphere to Plane Contact Force in Simscape Multibody Contact Forces library) was used to simulate the interaction effect between the robot foot and the ground and to produce the ground reactive force and horizontal friction force.

**Table 1:** Parameter values of the simulation model.

**Parameters**	**Mass m (kg)**	**Moment of inertia I (kg m^**2**^)**	**Length L (m)**
Torso	10.473	0.22	0.476
Upper leg	1.541	0.0447	0.426
Lower leg	1.154	0.019	0.502
Foot	0.691	0.00043	0.273
Leg length	-	-	0.996

### Controller

**Figure 2:**
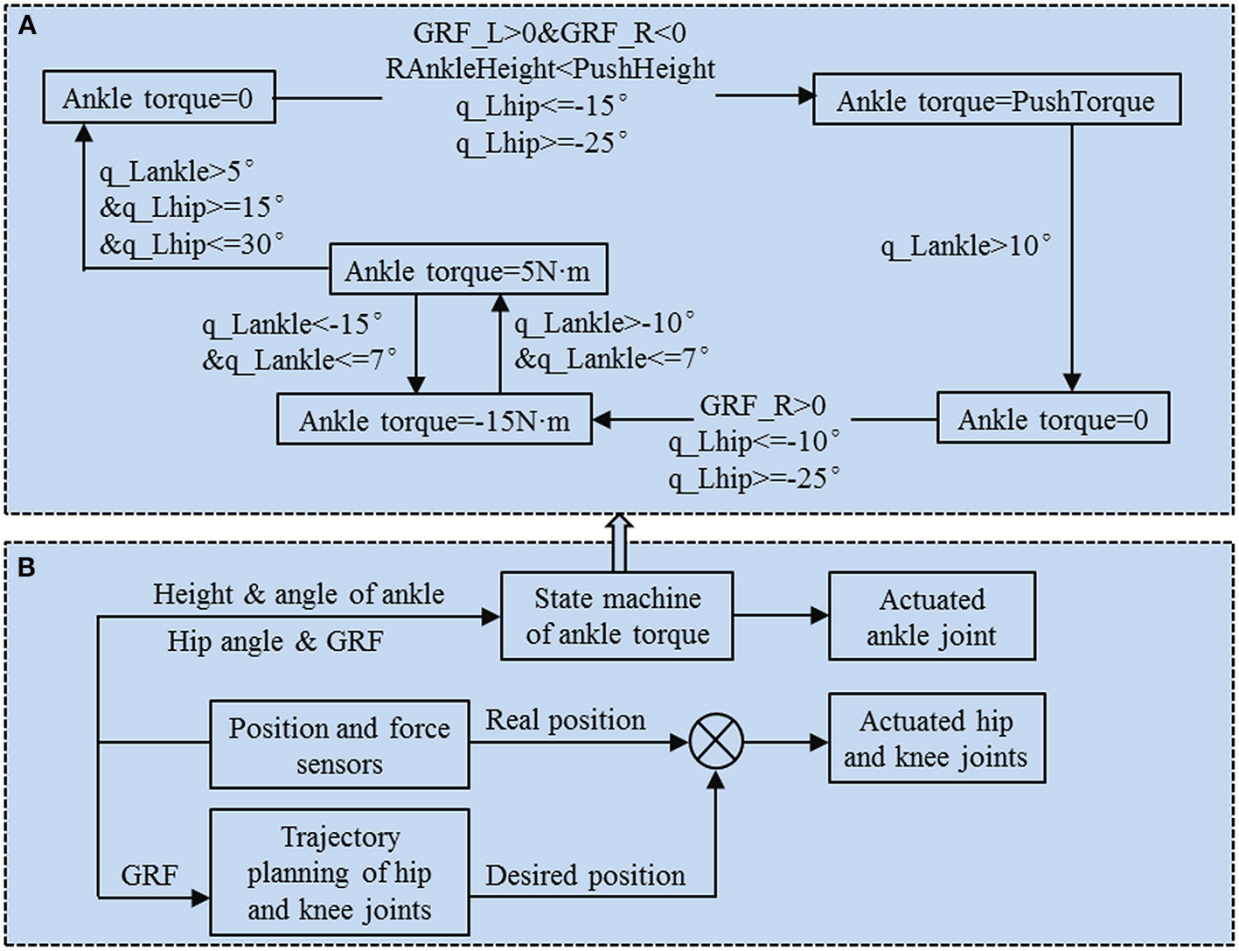
Block diagram of the controller system. **(A)** Ankle torque state machine flowchart. Ankle torques have four states. The state values of the torque are 0, push torque, −15 N·m and 5 N·m. Various state transfers were performed using the conditions of whether the foot touched the ground, the height of the ankle joint, the ankle angle and the hip angle. **(B)** Overall controller of bipedal robot.

It is well known that a biped robot is a hybrid dynamic system. A gait cycle consists of two phases. The stance phase is a continuous stage, and the process of a foot collision with the ground is a discrete phase (Garcia et al., 1998; Grizzle et al., 2014). Some studies have realized the hybrid system of bipedal robots by using a finite state machine (Pratt, 2000; Geng and Gan, 2010; Zhao et al., 2017). In this study, the finite state machine has also been used to control the cooperative motion between the joints of the robot. The details of the state machine of the hip and knee joint refer to previous study (Geng and Gan, 2010). The block diagram of the controller system is composed of the state machine of ankle torque and the overall controller of bipedal robot, as shown in Figure 2. The actuated hip and knee joints are controlled by the proportional–integral–derivative (PID) controller. The desired positions of hip and knee joints are obtained by using the module of trajectory planning. The real positions of hip and knee joints and the ground reaction forces (GRF) are acquired by using the module of position and force sensors, as shown in Figure 2B. The ankle joint is actuated by a hybrid method with an active actuated mode and a passive underactuated mode. When the condition of ankle push-off is satisfied, the ankle joint is actuated in the active mode; otherwise, it is underactuated in the passive mode. The ankle joint is determined to be in passive underactuated or active actuated mode according to the ankle torque state machine (see Figure 2A).

To test the effect of the ankle push-off torque and push-off height (the push-off height is the height of the ankle joint of the swing leg) on the robot's walking speed, the ankle torque state machine was designed to perform different ankle torques on the robot. Taking the left leg ankle torque state machine as an example, the states of the transfer processes of the ankle torque are explained in detail. As shown in Figure 2A, when the left leg touches the ground and the right leg does not touch the ground, the right leg hip joint is located within −25°~−15°, and the height of the right leg ankle joint is less than the push-off height. Then, the ankle push-off of the left leg is triggered, and the ankle joint is actuated in the active mode. When the left leg ankle angle was less th an10°, the ankle push-off disappeared. To prevent the foot from scuffing the ground during the swing period, the ankle torque state machine limits the ankle angle to the appropriate range. When the left hip joint is in the range of −25°~−10° and the right leg has touched the ground, the ankle torque is −15 N·m. When the left hip joint is less than 7° and the ankle angle is in the range of −15°~−10°, the ankle torque is converted between 5 and −15 N·m. When the left ankle angle is >5° and the left hip joint is in the range of 15°~30°, the ankle torque is 0 N·m. The ankle torque is converted to 0 N·m before the stance phase, and the ankle is underactuated in the passive mode at this time.

In the study, ankle push-off occurred before the heel strike, and the knee flexion of the trailing leg was determined by whether or not the leading leg touched the ground. Thus, the knee joint is in extension when ankle push-off of the trailing leg occurs. To prevent the phenomenon that impulsive ankle push-off leads to the catapult-like action of the robot during the knee is in extension (Lipfert et al., 2014), the impulsive ankle push-off torque acquired from the ankle torque state machine is modified to avoid the sudden increase in the torque resulting from the robot bouncing off the ground. The correction method of the ankle push-off torque is to plan a smooth fifth-order polynomial curve within 0.1 s of the initial push-off. The curve is smooth from the beginning to the end. Both the step length and the walking speed are affected by ankle push-off (Dean and Kuo, 2009), and the walking speed increases with the step length. This study aims to explore the effect of the torque magnitude of ankle push-off on the walking speed, so the step length is set to a fixed value. The literature (Zelik and Kuo, 2010) shows that the range of motion of the hip joint is approximately 40° during normal human walking speed. Therefore, the angle between the two legs of the robot is set to 40°. The initial position of the biped robot is that the left leg is the leading leg and the right leg is the trailing leg. The corresponding hip joints are 20° and −20°. The knee joints of both legs are −5° in extension. The left leg foot heel and the right leg foot toe touch the ground. In the planning trajectory, it takes 0.5 s for the single leg hip joint to move from 20° to −20° and the knee joint to move from the extension state of −5° to flexion state of −45°. To make the robot walk normally, we apply a thrust to the robot's torso at the initial state. The purpose is to make the robot's COM position exceed the highest point of the inverted pendulum so that it does not fall backward.

The biped robot simulation model is numerically calculated by using MATLAB's solver ode23. The GRF, the actual trajectory of the joint and the joint torque are obtained. The average COM speed in the horizontal direction is used as the walking speed of the biped robot. The simulation model biped robot has 133 components. The mass (mi) of each component is defined according to the material properties of the component. Every component's COM position (*xi, yi, zi*) relative to the global coordinate system is recorded by using the transform sensor in the Simscape toolbox. The total mass of the robot (M) is 17.26 kg. Thus, the COM position (*Xi, Yi, Zi*) of the robot is acquired by using the COM calculation formula. Then, the instantaneous COM speed of the biped robot is obtained by using the differential module in MATLAB Simulink. The simulated model can acquire the continuous walking for at least 30s.

### Simulation Experiment

This study imitates the effects of the impulsive ankle push-off in human walking and explores the influence of the ankle push-off torque and push-off height on the walking speed for the planar biped robot. Ankle push-off occurs in approximately 20% (45–65%) of the gait cycle during human walking, and the peak torque of the ankle is approximately 1.4 N·m/kg (Zelik and Kuo, 2010). The robot mass in this simulation is 17.24 kg and the reference value of the peak torque of ankle push-off is approximately 24 N·m. Thus, the range of the variable parameter ankle push-off torque is set as 16–626 N·m. The push-off height is measured from the ankle joint of the swing leg to the ground. When the foot completely touches the ground, the height of the ankle is approximately 6.68 cm. The range of the push-off height is set as 11–16 cm. The push-off height is roughly proportional to the gait cycle. Taking the average speed of the robot's COM as an index, the relationship between the push-off torque, the push-off height and the walking speed is tested.

The average speed of the COM, joint angle, and joint torque of the robot are mainly analyzed. The GRF is used to determine whether the foot touches the ground. One gait cycle is defined from the leg touch-down to the subsequent touch-down of the same leg, including the stance phase and the swing phase. The joint angle and joint torque are normalized to one gait cycle (0–100%), and the instantaneous walking speed within one gait cycle is normalized to the maximum walking speed of the gait. The normalized speed is calculated by the ratio of instantaneous speed to the maximum speed. In addition, to compare the ankle joint torque between humans and the simulation model at normal walking speeds, the ankle joint torque of the simulation robot is normalized to the body mass.

## Results

### Effect of the Ankle Push-Off Torque on the Walking Speed

**Figure 3:**
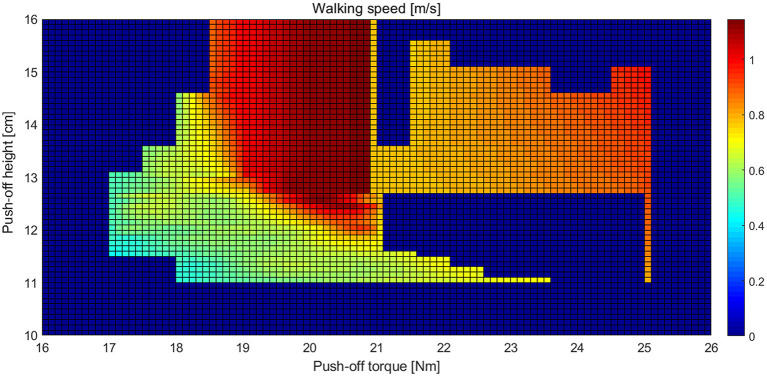
Landscape of the walking speed of the biped robot with various sets of the push-off torque and push-off height.

We studied the effects of the ankle push-off torque on the walking speed of the biped robot. As shown in Figure 3, when the ankle push-off torque is <20.8 N·m, the walking speed gradually increases as the push-off torque and push-off height increase. When the push-off torque is in the range of 21.5–25 N·m, as the push-off torque and push-off height increase, the walking speed gradually increases. When the push-off torque is <17 N·m or >25 N·m, no matter how the push-off height changes, the robot cannot walk normally. When the push-off torque is 20.8 N·m and the push-off height is >12.5 cm, the robot's walking speed achieves the maximum, approximately 1.14 m/s.

**Figure 4:**
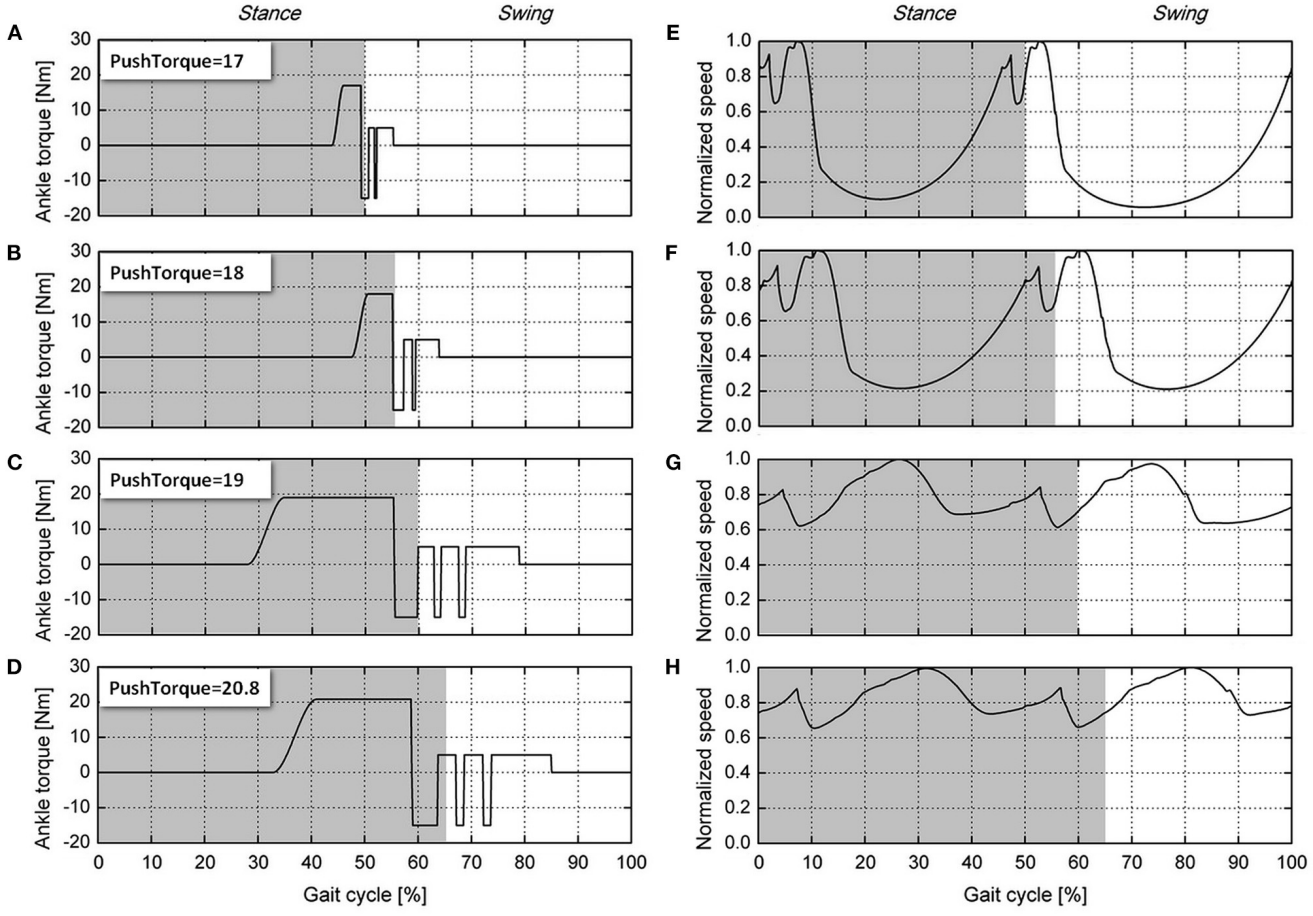
Ankle push-off torque and normalized walking speed during the gait cycle. The ankle joint push-off torques are shown: **(A)** Push torque is 17 N·m. **(B)** Push torque is 18 N·m. **(C)** Push torque is 19 N·m. **(D)** Push torque is 20.8 N·m. The normalized speeds are as follows: **(E)** push torque is 17 N·m, **(F)** push torque is 18 N·m, **(G)** push torque is 19 N·m, and **(H)** push torque is 20.8 N·m. The light gray areas indicate the stance phase, and the non-shaded areas indicate the swing phase.

To explore the reason that the robot's walking speed increases during the process of the push-off torque increasing from 17 to 20.8 N·m, when the push-off height is 13 cm, the relationship between the ankle push-off torque and the walking speed of the robot during one gait cycle is shown in Figure 4. Figures 4A–D shows that with an increasing ankle push-off torque, the stance period of the left leg gradually increases from 50 to 65%, and the swing period decreases from 50 to 35% of the gait cycle. Thus, the ankle push-off accelerates the movement of the swing leg and increases the walking speed of the robot, which is consistent with the conclusion proposed by Lipfert et al., i.e., the ankle push-off powers leg swing during human walking (Lipfert et al., 2014). In addition, when the ankle push-off torque is 17 N·m, as shown in Figure 4E, the normalized instantaneous speed changes from 0.05 to 1.0, with a wide range of fluctuation, which is 95% of the maximum speed. As the ankle push-off torque increases to 20.8 N·m, the normalized instantaneous speed changes from 0.65 to 1.0. The fluctuation range decreases to 35% of the maximum speed, as shown in Figure 4H. With an increasing ankle push-off torque, the fluctuation of the instantaneous speed is reduced, and the walking speed of the biped robot is increased. When the push-off torque is 17 N·m, ankle push-off occurs at 42–48% of the gait cycle, and the normalized speed increases from 0.57 to 0.66, as shown in Figures 4A,E. When the push-off torque is 18 N·m, the ankle push-off occurs at 47–56% of the gait cycle, and the normalized speed increases from 0.66 to 0.78, as shown in Figures 4B,F. When the ankle push-off torque is 19 N·m, the push-off occurs from 27 to 55% of the gait cycle, and the normalized speed shows a fluctuation trend that starts at 0.99 (27%), decreases to 0.69 (38%), increases to 0.84 (53%), and then decreases to 0.64 (55%), as shown in Figures 4C,G. When the push-off torque is 20.8 N·m., the ankle push-off occurs at 33–58% of the gait cycle. The normalized speed also shows a fluctuation from 0.98 (33%) to 0.74 (44%), then increased to 0.88 (57%) and next decreased to 0.75 (58%), as shown in Figures 4D,H. In summary, the ankle push-off helps to increase the COM speed of the robot during the walking gait.

**Figure 5:**
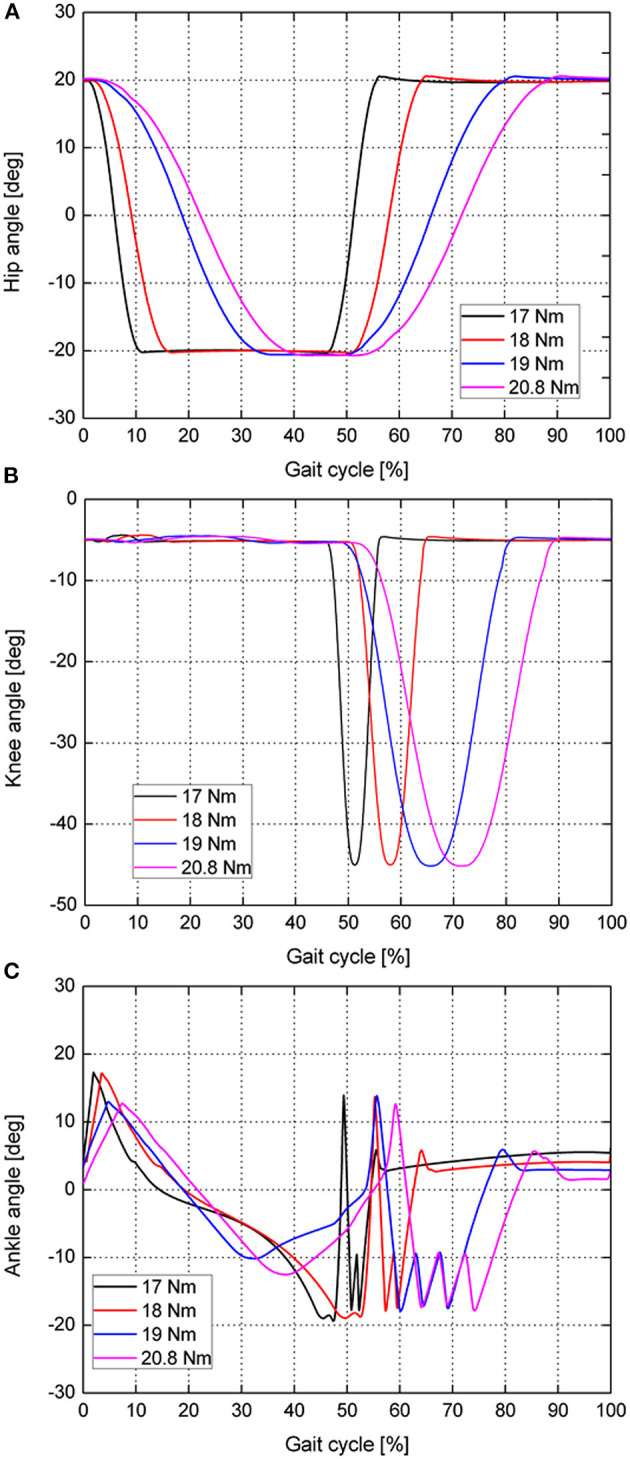
Robot joint kinematics during one gait cycle: **(A)** Hip angle. **(B)** Knee angle. **(C)** Ankle angle. The black line indicates the joint kinematics when the ankle push-off torque is 17 N·m. The red, blue and pink solid lines correspond to the joint kinematics when the ankle joint push-off torques are 18, 19, and 20.8 N·m, respectively.

Furthermore, the joint kinematics are obtained at different ankle push-off torques (17, 18, 19, and 20.8 N·m, respectively), as shown in Figure 5. It can be seen from Figures 5A,B that the movement patterns of the hip and knee joints are basically similar. As the ankle push-off torque increases, the hip moves slower from 20° to −20°, and the corresponding period increases from 10 to 40% of the gait cycle. Under the condition of different ankle push-off torques, the timing difference of the knee starting to flex is small, but the timing difference of the knee joint finishing the flexion and extension is large, as shown in Figure 5B. When the ankle push-off torque is 17 N·m, the duration accounts for 10% of the gait cycle for the knee joint to move from the flexion to the extension. When the push-off torque increases to 20.8 N·m, the duration accounts for 40% of the gait cycle for the knee joint to move from flexion to extension. This result also shows that the movement of the knee joint is consistent with the movement of the hip joint, as shown in Figures 5A,B. The motion curve of the ankle joint is shown in Figure 5C. At the early stance phase, the ankle extends approximately 15° after touch-down until the complete foot is in contact with the ground. With the foot entirely in contact with the ground, the body moves forward with the shank rotating around the ankle joint. The ankle begins to flex, and the joint angle gradually decreases. Until the ankle push-off conditions are satisfied, the ankle torque begins to change from a passive underactuated mode to an active actuated mode. At this time, the push-off torque is applied to the ankle, so the ankle starts to extend and the ankle angle starts to increase, as shown in Figure 5C. When the ankle angle increases to 10°, the ankle push-off torque becomes 0 N·m, the ankle begins to flex to −17°, and the left leg starts to swing. To prevent the foot from scuffing the ground, the ankle angle is controlled in the range from −15° to −10° by using an ankle torque state machine (Figure 3). When the swing leg moves to the front of the body, i.e., the hip angle >15° and the ankle angle >5°, the ankle joint torque becomes 0 N·m. The ankle joint push-off torque is switched from the active actuated mode back to the passive underactuated mode in preparation for the next touch-down.

### Gait Comparison Between the Simulation Robot and Human

**Figure 6:**
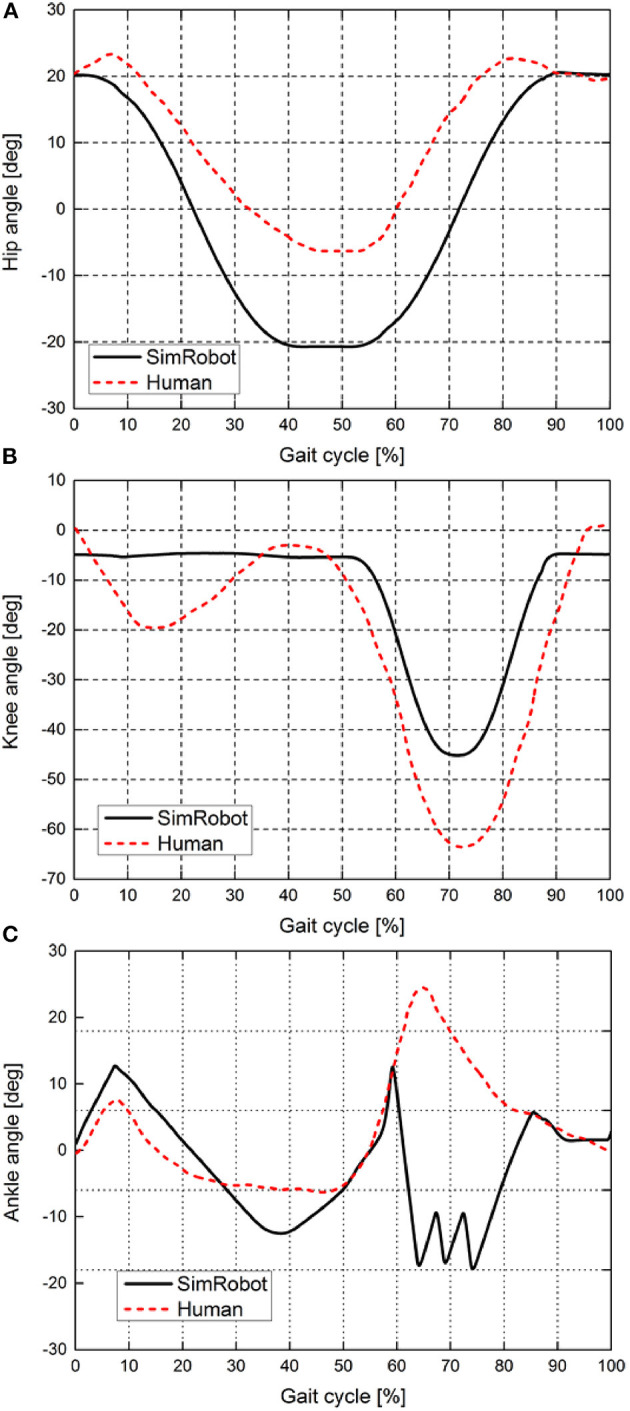
Joint kinematics of the simulation robot and a human at a normal walking speed. **(A–C)** show the motion curves of the hip, knee, and ankle joints, respectively. The black solid line indicates the joint kinematics of the simulation robot, and the red dashed line indicates the joint kinematics of the human during normal walking speed.

To study whether the ankle push-off of the robot had a positive effect on walking speed, similar to the role of ankle push-off during human walking, the joint kinematics and ankle torques of the simulated robot at 1.14 m/s are compared with the human data at the normal walking speed (1.04 m/s). The human data are taken from the literature (Geng and Gan, 2010; Lipfert, 2010). The size parameters of bipedal robot are similar to the adult humans. Figure 6 presents the joint angle comparison between the simulation robot and human at a normal walking speed. The results show that the hip and knee joints of the simulation robot and human have similar motion patterns at a normal walking speed. The range of motion of the hip joint of the simulation robot is larger than that of humans (Figure 6A), but the range of motion of the knee joint is smaller than that of humans (Figure 6B). Notably, the motion pattern of the ankle joint of the simulation robot is similar to that of a human. Two peak waves of the ankle angle are observed during walking of the simulation robot, i.e., the robot touch-down and the ankle push-off (Figure 6C). The ankle push-off of the simulation robot appears at 33–58% of the gait cycle, and the knee joint is basically kept straight. While the ankle push-off of the human occurs later than that of the simulation robot, it occurs at 45–65% of the gait cycle, and the knee joint is in a flexed state.

**Figure 7:**
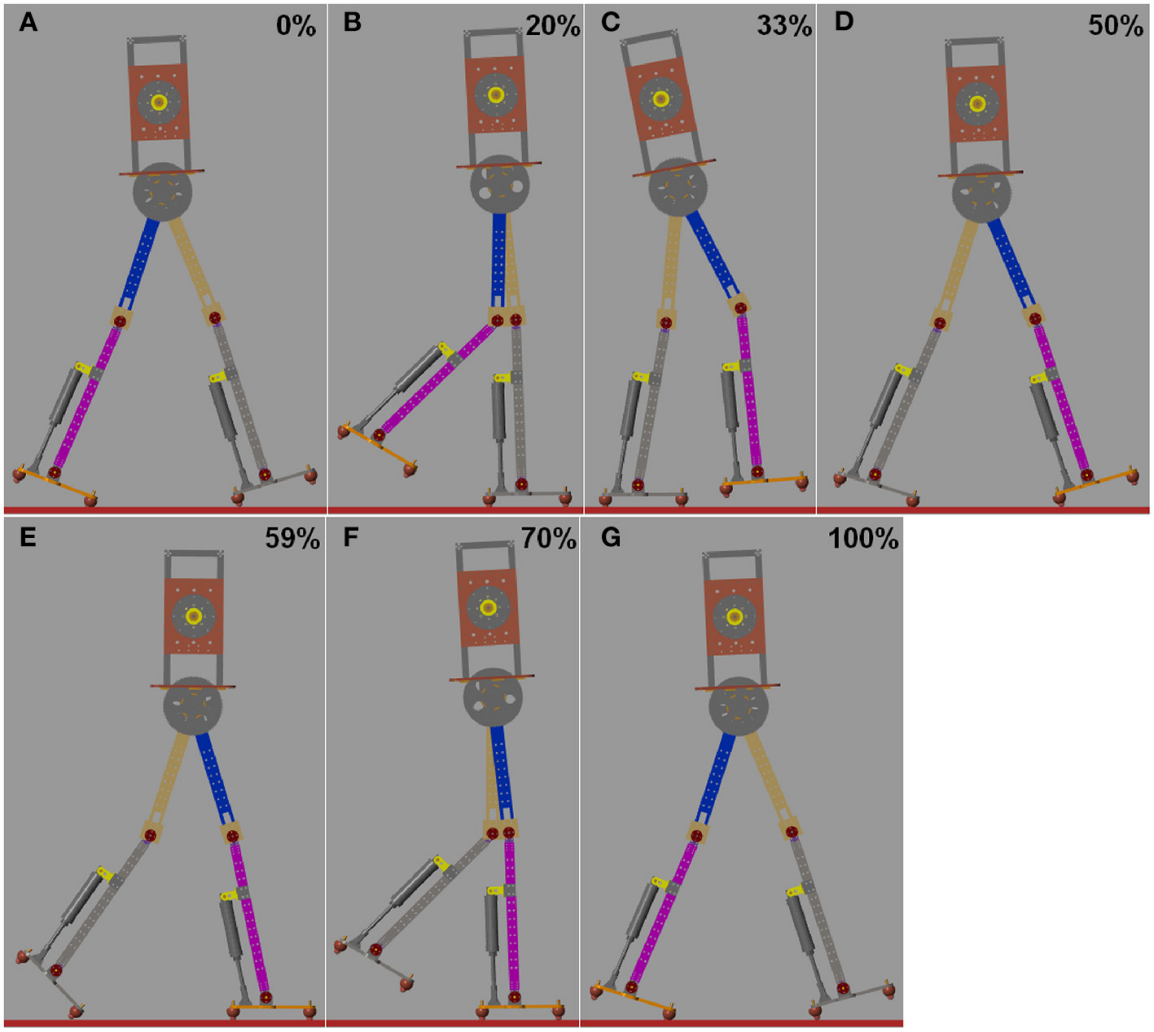
Series of frames of the simulation robot during one gait cycle at 1.14 m/s. **(A)** Touch-down at 0%. **(B)** The whole foot is in contact with the ground. **(C)** Beginning of the ankle push-off at 33%. **(D)** During ankle push-off. **(E)** Ending of the ankle push-off. **(F)** Swing phase. **(G)** The swing leg touches the ground and a next step begins.

Figure 7 shows snapshots of the simulation robot during one gait cycle when the ankle push-off torque is 20.8 N·m (see Video S1). The initial state is that the left heel touches the ground and the right toe touches the ground, as shown in Figure 7A. As the body moves forward, the left foot is complete in contact with the ground. At 33% of the gait cycle, the left leg ankle joint begins to push off, as presented in Figure 7C. At 59% of the gait cycle, the left leg ankle joint push-off ends (Figure 7E). After that, the left leg starts to swing until the heel touches the ground, preparing for the next step.

**Figure 8:**
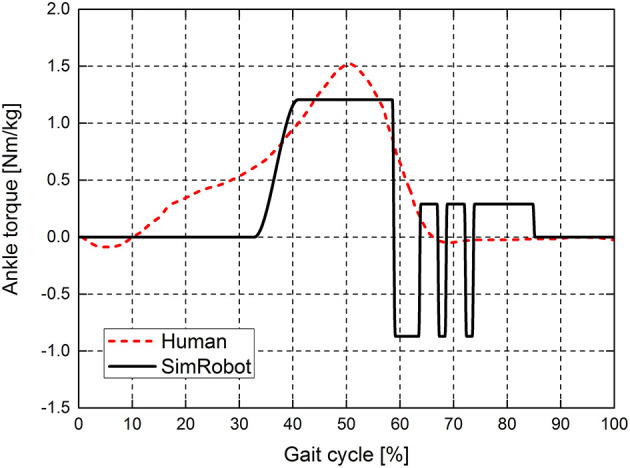
Ankle joint torques of the simulation robot and human at a normal walking speed. The ankle joint torque is normalized to the body mass. The red dotted line indicates the ankle joint torque of the human, and the solid black line indicates the ankle joint torque of the simulation robot.

In order to fairly compare the ankle torque between the simulated biped robot and humans, we use the normalized ankle torque, ankle torque divided by the body mass. The normalized ankle joint torque of the simulation robot and human at a normal walking speed are shown in Figure 8. At the early stance phase, the ankle torque decreases as the ankle extends during human walking. As the body moves forward and the whole foot contacts with the ground, the ankle torque increases. Then, ankle push-off occurs at the end of the stance phase. When the toe lifts off the ground, ankle torque starts to decrease at 50% of the stance phase. Then, the leg starts to swing, and the ankle torque decreases to 0 N·m. When the push-off torque is 20.8 N·m and the push-off height is 13 cm, the ankle torque of the simulation robot and human have similar patterns during normal walking speed.

## Discussion

In this study, the simulation robot achieves a maximum walking speed of 1.14 m/s. The leg length of the simulation model is 0.996 m, as shown in Table 1. Then, the dimensionless speed (the normalized Froude number *Fr*) is 0.36. “Meta” obtains a maximal speed of 0.68 m/s (the maximum *Fr* = 0.28) by adjusting the amount of ankle push-off or upper body pitch. Compared with the walking speed of the biped robot “Meta,” the speed of simulation robot is greater than that of “Meta” (Hobbelen and Wisse, 2008b). Additionally, the passive knee joint of “Meta” limits a further increase in the walking speed. Renjewski and Seyfarth (2012) explored the effect of ankle push-off on robot walking and showed that the ankle movement pattern is significantly different from that of humans (Renjewski and Seyfarth, 2012). The ankle angle of the human data during a normal walking speed increases first at 50% of the gait cycle and then decreases at 65% of the gait period. However, the ankle angle in the literature (Renjewski and Seyfarth, 2012) basically remains stable at the late stage of the gait cycle. The compliant ankles with soft bidirectional rotational springs were used to push off the biped robot. Because the springs cannot provide a sufficient driving torque for the ankle, the movement mode of the ankle joint of the robot is also quite different from that of humans. It also further suggests that the passive ankle push-off does not effectively provide the ankle joint with a similar movement pattern to humans. Our results show that the active actuated ankle push-off can increase the walking speed of robots and that the ankle angle presents a similar pattern to the human data at 0~60% of gait cycle, as shown in Figure 6C. During 60~85% of gait cycle, the motion pattern of ankle joint of simulated biped robot is different from that of human beings. This is due to that the discrete control by using finite state machine produced discrete ankle torque at 60~85% of gait cycle, as shown in Figure 8.

In this paper, the ankle push-off torque is similar to the impulse mode acquired from an ankle joint torque state machine, which is different from the continuous ankle torque curve of a walking human. However, it is demonstrated that a simple and impulsive ankle push-off strategy can increase the walking speed by accelerating the swinging leg under the appropriate push-off torque and push-off height. The results suggest that when the ankle push-off torque is more than 20.8 N·m, the walking speed does not increase with increasing push-off torque. This is due to that the excessive push-off torque causes the trailing leg to swing too fast and the COM of the robot quickly moves to the front of the body. The trailing leg touches the ground in advance, and eventually, the robot falls forward.

In addition, the ankle push-off of the simulation robot occurs earlier than that of humans during a normal walking speed. During the phase of ankle push-off, the knee of the simulation robot is basically straight, and the knee of the human is in a bending state. This is due to that the multiple joints of the simulation robot cannot transmit the force and torque as effectively as the human lower limb cross joint muscles and tendons. There is no good synergy between the joints of the simulation robot. In this paper, the movements of the knee joint and the hip joint are synchronized. If ankle push-off occurs after knee bending, then the hip joint also moves forward as the knee bends. Thus, it is difficult to generate a sufficient GRF between the foot and the ground. The GRF generated by the ankle push-off also cannot be effectively transmitted to the robot torso. Simulation results also demonstrate that when ankle push-off occurs after knee bending, robots may even fall backward. Therefore, the ankle push-off after knee bending has a small contribution to the walking speed of the simulation robot. However, the human lower limb is a complex rigid-flexible coupling system consisting of bones, muscles, and tendons. Multiple joints can transmit force and torque using muscles and tendons (Zelik and Adamczyk, 2016). During human walking, even if the knee is bending, the ankle push-off torque can be transmitted to the torso through the muscles across the joint to push the body forward.

Zelik and Adamczyk (2016) showed that the ankle push-off primarily contributes to both leg swing and COM acceleration during human walking (Zelik and Adamczyk, 2016). In this study, the ankle push-off also increases the COM speed for the simulation robot. Saunders et al. suggested that ankle push-off smooths the COM trajectory during the step-to-step transition (Saunders et al., 1953). With the increasing torque of the ankle push-off, the fluctuation COM speed is reduced and becomes smooth, which contributes to an increase in the walking speed of the simulation robot.

Finally, to compare with human walking, in this study, the angle between two legs is set to a fixed value (40°), and the motion trajectory of the hip is also planned. Only the effects of ankle push-off on walking speed are tested in the simulation model under the above conditions. The simulation is a very powerful tool in robotics and it can be used to design different control algorithms, to design mechanical structure of robots, to design robotic cells, etc. Furthermore, using the simulation tools one can avoid injuries and damages, and shorten the development cycle and manufacturing process of production (Žlajpah, 2008). In this study, the simulation results and parameters of the simulation robot model may provide a reference for future prototype tests. For the bipedal robots of different size and mass, the researchers can acquire the roughly scope of parameter values of push-off height and ankle torque during push-off stage by using our simulation methods. However, the simulation has some limitations, e.g., the motor models and fast collision detection are not accurate enough as the real environment. Therefore, in the future, the effect of the ankle push-off torque on the walking speed will be evaluated in a biped prototype. The ankle will be actuated by a pneumatic cylinder in the biped prototype. In addition, in the study, the ankle joint push-off torque mode is relatively simple, so the walking speed may be considered to be the optimization goal to obtain the complicated curve of the ankle push-off torque that is suitable for biped robots.

## Conclusion

In this study, we have explored the effect of the impulsive ankle push-off torque on the walking speed of a planar bipedal robot in simulation. The results show that when the push-off height of the ankle joint is 13 cm and the ankle push-off torque increases from 17 to 20.8 N·m, the duration of the swinging leg actually decreases from 50 to 30% of the gait cycle, the fluctuation amplitude of the COM instantaneous speed of the robot decreases from 95 to 35% of the maximum speed, and the walking speed increases from 0.51 to 1.14 m/s. In addition, when the walking speed of the simulation robot is close to the normal walking speed of the human, it shows that the joint motion pattern of the simulation robot is similar to that of the human, and the mode of ankle push-off torque is also basically similar. However, the push-off timing of the simulation robot (56% of stance period) is earlier than that of the human (69% of stance period). Finally, the simulation results demonstrate and confirm that ankle push-off is an effective method to increase the walking speed of the biped robot.

## Data Availability Statement

The raw data supporting the conclusions of this article will be made available by the authors, without undue reservation.

## Author Contributions

QJ was responsible for the simulation and manuscript preparation. ZQ and LeR participated in discussions and revisions. LuR is involved in funding acquisition and worked as supervisors for all procedures. All authors contributed to the article and approved the submitted version.

## Conflict of Interest

The authors declare that the research was conducted in the absence of any commercial or financial relationships that could be construed as a potential conflict of interest.

## Supplementary Material

Video S1 When the ankle push-off torque is 20.8 N.m, the video of biped robot at fast walking speed (1.14 m/s) is presented.
